# From Concept to Perception: Equestrian Definitions of Harmony and Visual Attention in Horse–Rider Evaluation

**DOI:** 10.3390/ani16101483

**Published:** 2026-05-12

**Authors:** Inga A. Wolframm, Madita Everding, Varvara Savulchyk, Jorinde Borssen, Debby D. M. Gudden

**Affiliations:** Applied Research Centre, Van Hall Larenstein University of Applied Sciences, 6880 GB Velp, The Netherlands

**Keywords:** harmony, visual attention, eye tracking, equestrian sport, perception, judging criteria, horse–rider interaction, performance evaluation

## Abstract

In equestrian circles, horse–rider harmony is understood intuitively, yet clear criteria are lacking. This study examined how different equestrians conceptualized and visually assessed harmony and how this influenced scoring. Qualitative interviews were combined with eye tracking technology. Thirty equestrians assessed five videos of horse–rider combinations performing in dressage, showjumping, eventing, working equitation and Icelandic riding, with eye movements being recorded using a mounted eye tracker. Verbal definitions were analyzed using thematic analysis, revealing three overarching themes: Horse Behavior, Horse–Rider Connection, and Rider Influence. Number of fixations and duration of fixation were reduced using principal component analysis (PCA), yielding five components each. These explained 70.9% and 64.5% of variance, respectively. Linear mixed-effects models showed significant effects for two PCA components: frequent fixation on the horse’s ears and eyes relative to the horse’s shoulder and rider leg predicted lower harmony scores (B = −0.34, SE = 0.13, z = −2.53, *p* < 0.05), whereas longer dwell time on the rider’s shoulder relative to the rider’s leg predicted higher scores (B = 0.25, SE = 0.12, z = 2.12, *p* < 0.05). Ears and eyes were also the most frequent first fixation. Harmony appears to be a shared construct at the conceptual level, but is personally enacted at the practical level. Equine facial expressions and rider posture serve as important perceptual indicators.

## Simple Summary

Most equestrians think they can recognize a harmonious horse–rider combination when they see it. Yet, what precisely they look at and why remains unclear. This study explored how different equestrians conceptualize harmony, what they look at when assessing it, and how this might influence the scores they give. Thirty participants watched five short videos of horse–rider combinations performing in dressage, jumping, eventing, working equitation and Icelandic riding while their eye movements were recorded. They also described what harmony meant to them. These descriptions were summarized into three categories: Horse Behavior, Horse–Rider Connection, and Rider Influence. Eye tracking results also showed that participants used distinct visual search strategies but only two of these influenced harmony scores. First, the horse’s ears and eyes compared to the horse’s shoulder and rider leg, and second, the rider’s shoulder compared to the rider’s leg. Ears and eyes were also looked at first most often. These findings suggest that harmony is a broadly shared concept, but that its practical application varies considerably between individuals. The horse’s facial features and rider position stand out as important visual clues.

## 1. Introduction

Harmony has been valued across cultures as a symbol of balance and well-being. In ancient Greece, philosophers such as Pythagoras and Plato described it as a form of balance and justice [1]. Similarly, in Chinese philosophy, harmony is seen not as the absence of tension, but as a dynamic process of balancing opposing forces [2,3]. This notion often extends into human–animal relationships, including those between humans and their horses [4]. Many, if not all equestrian sports, celebrate the unique bond between the horse–rider dyad, which is built through time and mutual understanding [5,6]. More specifically, the intuitive and refined communication developed through experience and ongoing interaction between horse and rider is often referred to as “the feel” [6] and is considered essential to both performance and welfare [7].

In the context of equestrian sports, therefore, harmony is more than a philosophical ideal. It is also a practical and evaluative concept, deeply embedded in equestrian culture. In the discipline of dressage, it is assessed as one of five artistic marks [8]. Similarly, in Icelandic riding harmony is considered essential for the score in the category Riding Skills/Connection [9], while working equitation scores include horse–rider harmony during the obstacle course [10].

Yet, despite these formal inclusions in judging guidelines, harmony remains poorly defined and currently relies on human perceptual interpretation. Over the years, researchers have proposed a range of definitions and indicators of harmony. Biomechanically, harmony has been operationalized as the consistency and regularity of coupled motion between horse and rider. Peham et al. [11] quantified it as the regularity of angular relationships between rider and horse in phase space, finding that professional riders produced significantly more consistent movement patterns than recreational riders, correlating directly with higher dressage scores. Lagarde et al. [12] extended this using a coordination dynamics framework, demonstrating that expert riders maintain continuous phase synchronization with the horse’s oscillatory movements through haptic contact, while novices show transient departures from synchrony. Wolframm et al. [13] similarly demonstrated that movement coupling in horse–rider dyads varied systematically with gait, with canter showing greater levels of synchronization than other gaits, underscoring the importance of biomechanical coupling as a precondition for the effective, non-interfering communication of aids. More recently, Hobbs et al. [14] used inertial sensors to evaluate performance in high-level dressage combinations, finding meaningful relationships between objectively measured movement parameters and general impression scores awarded by judges. Uldahl et al. [15] further highlighted the rider’s physical contribution, demonstrating that pelvic mobility and balance were associated with equestrian skill level and had implications for horse welfare.

Psychologically, harmony has been associated with the rider’s development of an intuitive feel, described as reflecting mutual understanding, commitment, and a sense of a perfect match between horse and rider [6]. Tufton and Jowett [6] identified closeness, trust, mutual responsiveness and empathic accuracy as the psychological foundations of this relational quality in elite riders’ accounts, underpinning both performance and welfare. Wolframm and Meulenbroek [16] further demonstrated that the self-perceived personality traits of riders co-varied with perceptions of horse temperament, suggesting that psychological compatibility forms part of how people experience and evaluate horse–rider interaction quality. Hogg and Hodgins [17] similarly found that elite riders positioned the horse–rider relationship in complex and sometimes contradictory ways in relation to sporting outcomes, with harmony in dressage judging emerging as a persistent source of ambiguity.

More recently, researchers have investigated physiological mechanisms underpinning horse–rider synchronization. Callara et al. [18] drew on heart rate variability synchronization to examine horse–human interactions across different experimental conditions, finding that both horse and human responded physiologically and that familiarity between the two shaped the strength of the interaction. Similarly, Helmer et al. [19], examining equine-assisted therapy with children, found evidence of bi-directional heart rate synchronization between horses and riders, with synchronization strength influenced by rider experience. While these studies differ in context from competitive equestrian sport, they collectively suggest that horse–rider synchronization operates across multiple physiological and psychological channels.

At the same time, conflict behaviors in the ridden horse, such as tail swishing or excessive mouth activity are widely observed in competition footage and are associated with discomfort, miscommunication, or welfare concerns [5,20]. These behaviors can result, among other things, from inconsistent rider cues and poor balance [5,21]. Although some signs are explicitly penalized, others are inconsistently judged, and in some cases even rewarded, despite being inconsistent with official guidelines [8,20]. This inconsistency poses not only welfare concerns but also risks the sport’s social license to operate, defined as continued public acceptance of an activity based on real and perceived animal welfare standards [22,23].

In competitive settings, the recognition and evaluation of such behaviors ultimately depend on judges’ interpretations. Judging decisions are typically made under time pressure and cognitive load, requiring quick interpretation of complex visual information without the opportunity for replay or review [24,25,26]. In such contexts, judges rely on heuristics, i.e., cognitive shortcuts that help manage information overload which, while efficient, can introduce systematic biases and reduce consistency [26,27]. Seeing that judging in equestrian sport is fundamentally a visual task [28], these heuristics are closely linked to how judges allocate their visual attention: salient cues are likely to draw attention quickly, while subtle signals of discomfort may go unnoticed [20,25].

As visual information can only be processed during brief periods of relative gaze stability referred to as fixations, examining how judges or other equestrian practitioners distribute their gaze can offer valuable insights into how perceptual processes shape decision-making [25,28,29]. Eye tracking studies across sports have shown that expert judges demonstrate more efficient visual search strategies than novices, often including earlier and more frequent fixations on performance-relevant features [30,31,32]. This suggests that expertise is associated with refined visual search strategies that enable judges to filter irrelevant information and selectively focus on relevant cues [33]. In equestrian disciplines, previous research has shown that judges tend to focus more on the horse’s front, such as the head–neck position, with attention patterns varying by experience level and movement type [13,28]. It remains unclear, however, how such patterns of visual attention align with the conceptualization of horse–rider harmony, and how this might be reflected in evaluations more broadly, whether in formal competitive scoring or in the everyday assessments made by riders, trainers and educators. A clearer understanding of how harmony is perceived and assessed is essential for promoting transparent and consistent assessments, as well as equine welfare [20,26,34] both within the industry and in the wider public.

The current study therefore aimed to examine how equestrians with different levels of expertise conceptualize and visually assess horse–rider harmony, and how these processes influence numerical harmony scores as a proxy for evaluative decision-making.

## 2. Materials and Methods

### 2.1. Study Design

The study employed a mixed-methods design utilizing qualitative interviews and quantitative eye tracking analysis. Ethical approval was granted by the ethics committee of Van Hall Larenstein University of Applied Sciences (Dossier Number 004).

### 2.2. Participants

In order to capture a broad spectrum of equestrian perspectives, participants were recruited opportunistically at different equine events and locations in March and April 2024. A total of 57 participants were recruited at the equestrian fairs Equitana Germany (*n* = 18), Dutch Masters (*n* = 5), and Equiday (*n* = 15), as well as at the Van Hall Larenstein University of Applied Sciences (*n* = 9) and an Icelandic judging seminar (*n* = 11).

Participants were divided into two groups: amateur horse owners and riders (‘Amateurs’), and those working in the equestrian sector in a (semi)-formal capacity, including judges, trainers, teachers, and journalists (‘Professionals’). Participants provided written informed consent after being informed of the aims of the study and their right to withdraw at any time. Following initial data screening, 27 recordings were excluded, resulting in an exclusion rate of 47%. Exclusions were due to two technical data quality issues: insufficient gaze quality resulting from suboptimal lighting conditions at the data collection venues, and inaudible audio recordings affecting the qualitative data. Both are inherent challenges of conducting mixed-methods research in applied field settings rather than controlled laboratory conditions. In line with standard practice, recordings that did not meet minimum quality thresholds were excluded rather than retained, as including poor quality data would have introduced greater measurement error than exclusion. As recruitment was opportunistic and exclusions were based solely on technical quality criteria unrelated to participant characteristics or viewing behavior, systematic bias in the retained sample is considered unlikely. The final sample for analysis consisted of 30 participants (judges, *n* = 8; trainers, *n* = 9; amateur riders, *n* = 4; horse owners, *n* = 6; teachers in equine education, *n* = 2; journalists, *n* = 1). 

### 2.3. Data Collection

#### 2.3.1. Experimental Set-Up

Participants were asked to assess harmony levels from five pre-recorded, silent video clips of different horse–rider combinations performing in dressage, showjumping, eventing, working equitation, and Icelandic riding. The video clips ranged in length from 14.3 s to 19.0 s and depicted one discipline-specific movement each, performed at an intermediate level. Clip length was determined by the duration of one complete movement sequence in each discipline, and was kept brief to reflect the time-pressured conditions of real-world equestrian assessment and to minimize participant burden during data collection at busy equestrian events. For dressage, this included a left-hand canter with two flying changes; showjumping: three consecutive jumps at 1.30 m in height, including canter strides in between; eventing: the approach, take-off, and landing of a bank complex; working equitation: a slalom with flying changes for each change of hand; Icelandic riding: one long side performed at a tölt. All riders depicted in the videos provided explicit consent. Due to the limitations of the system, all video clips were presented in the same predetermined order.

#### 2.3.2. Eye Tracking

Participants’ eye movements were recorded using a Tobii Pro Fusion eye tracker (Tobii, Stockholm, Sweden), mounted on a 15″ Lenovo ThinkPad T480 (Lenovo, Beijing, China), sampling at 120 Hz. The velocity threshold for the fixation filter was set at 30 degrees/s, which is appropriate for handling eye tracking data in controlled studies with minimal head movement. Verbal comments were recorded and transcribed using Microsoft Teams. At the start of the experiment, the eye tracker was calibrated for each participant according to the manufacturer’s guidelines.

Following calibration, participants were shown two introductory slides outlining the purpose of the study and providing instructions on scoring different horse–rider combinations based on harmony. Having read the information, participants pressed Enter to view the first video clip. After each clip, participants were asked to verbally rate the horse–rider combination on harmony on a 0–10 scale (‘score’). Scores were recorded manually by the second author. After all videos were scored, participants provided a verbal definition of harmony in response to the open-ended prompt, “What does harmony mean to you?”. This sequence, i.e., video scoring first, followed by verbal definitions, was chosen to allow participants to engage with the visual assessment task naturally, drawing on their existing experiential knowledge without conceptual priming. The verbal definitions therefore reflect the crystallization of tacit knowledge activated by direct exposure to the stimulus material, consistent with established approaches in expert cognition research, where domain-specific knowledge is often most accurately accessed through reflection following task engagement [6].

### 2.4. Data Processing and Analysis

#### 2.4.1. Areas of Interest (AOIs)

**Figure 1:**
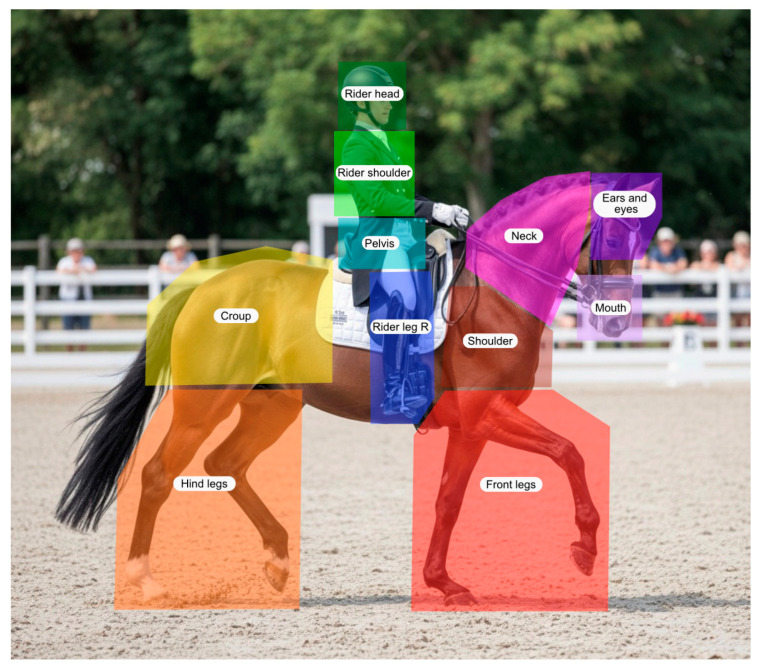
Example of how anatomical areas of the horse and rider were predefined as per the 11 areas of interest (AOIs).

To analyze visual attention, 11 areas of interest (AOIs) were defined in Tobii Pro Lab (v24.21), representing anatomical regions of the horse–rider combination that may be relevant when judging harmony. These AOIs comprised the horse’s Ears and Eyes, Mouth, Neck, Horse’s shoulder, Front legs, Croup, and Hindlegs, as well as the Rider head, Rider shoulder, Rider pelvis, and Rider leg. To illustrate the placing of the AOIs while preserving participants’ anonymity, an illustrative base image was generated using Gemini (Version 3 Flash using the Nano Banana 2 image model; Google, Mountain View, CA, USA), onto which the AOIs were added by the authors (Figure 1).

For each AOI, the number of fixations (NOFs), duration of fixation (DOF), total fixation time (TFF), and order of fixation (OOF) were extracted for further analysis. 

#### 2.4.2. Quantitative Analysis of Eye Tracking Data

Eye tracking data were subsequently processed using Python (Version 3.12.13, Python Software Foundation, Wilmington, DE, USA) via Google Colab (Google LLC, Mountain View, CA, USA). Data manipulation and statistical modeling were conducted utilizing the pandas, numpy, scikit-learn, factor_analyzer, and statsmodels libraries. Variables relating to gaze behavior and qualitative theme coding were converted to a numeric format, with gaze counts and coded theme frequencies treated as zero where absent. At the AOI level, the final dataset yielded 1650 observations (30 participants × 5 videos × 11 AOIs). For all multivariate gaze analyzes, data were aggregated to the participant-by-video level, resulting in 150 observations.

To control for the natural variation in eye tracking behavior and recording length, fixation proportions and duration proportions were calculated for each participant–video observation by dividing AOI-specific counts and durations by the total number and total duration of fixations recorded within each video. Descriptive checks confirmed that both fixation and duration proportions summed to 1.00 for each observation.

#### 2.4.3. Qualitative Coding and Integration with Eye Tracking Data

Audio recordings were transcribed verbatim using Microsoft Teams. Dutch and German transcripts were translated into English using ChatGPT (Version 4o; OpenAI, San Francisco, CA, USA) and subsequently checked for accuracy by the first and third authors. An inductive thematic analysis was conducted in NVivo (Version 15; Lumivero, Denver, CO, USA). Initial open coding grouped recurring references to harmony into lower-order codes, which were then clustered into higher-order themes.

To integrate the qualitative and quantitative datasets, coded theme frequencies were collapsed to the participant level and converted into proportions. Because the proportions of the higher order themes came to 1.00 for each participant, not all themes were included simultaneously in exploratory inferential models to prevent perfect multicollinearity.

#### 2.4.4. Principal Component Analysis of Gaze Strategies

To identify broader visual inspection strategies, separate principal component analyses (PCA) were conducted on fixation proportions and fixation duration proportions across the 11 AOIs. Prior to PCA, AOI variables were standardized using z-scores to account for differences in scale and variance. The use of proportional AOI measures introduces inherent interdependence between variables, as increases in one region necessarily correspond to decreases elsewhere. While this approach was chosen to account for individual differences in overall fixation behavior, it results in correlation structures that may be close to singular, which limits the applicability of certain factorability diagnostics and requires cautious interpretation of component structure. PCA components were extracted using the eigenvalue > 1.0 criterion and subsequently rotated using Varimax rotation to aid interpretability. Rotated component loadings greater than 0.3 were considered to represent meaningful contributions to component structure. Visual strategy component scores for gaze fixation (FRC) and gaze duration (DRC) were calculated for each observation by projecting the standardized AOI data onto the rotated component matrix. 

#### 2.4.5. Correlations Among Gaze Strategies

Pearson’s r correlations were calculated within the participant-level fixation strategy scores, within the duration strategy scores, and across fixation and duration strategy scores. These analyses were used to assess the extent to which fixation-based and duration-based component structures reflected shared or distinct aspects of visual attention.

#### 2.4.6. First Fixation Attentional Orientation

Initial attentional orientation was identified using the first AOI fixated (OOF = 1). First fixations to croup and hindlegs were collapsed due to infrequency.

#### 2.4.7. Linear Mixed-Effects Modeling

To assess whether visual gaze strategies or first fixations were associated with harmony evaluations, linear mixed-effects models (LMMs) were estimated using restricted maximum likelihood (REML). Harmony score served as the continuous dependent variable, and participant identity was included as a random intercept to account for repeated observations. Discipline of the stimulus video (Reference: Dressage) and participant experience level (Reference: Amateur) were included as fixed covariates across all models. To evaluate the predictive utility of the visual strategies, a primary baseline model simultaneously evaluated all extracted fixation and duration PCA components.

Finally, to explore the integration of qualitative and quantitative datasets without risking model overfitting, participant-level qualitative theme proportions were assessed in additional exploratory LMMs. To maintain an appropriate ratio of observations to estimated parameters, the qualitative themes were modeled separately: once alongside the extracted fixation components, and once alongside the extracted duration components. To supplement inferential findings, marginal and conditional R^2^ values were calculated for all primary mixed-effects models following Nakagawa and Schielzeth [35], providing estimates of variance explained by fixed effects alone, and by the combined fixed and random effects structure, respectively.

## 3. Results

### 3.1. First Fixation

The horse’s Ears and Eyes was the most frequent first fixation location with 46 occurrences, followed by the Rider shoulder (28 occurrences) and the Rider pelvis (19 occurrences). A LMM predicting harmony scores based on the first fixated AOI revealed no significant main effects for any starting location (all *p*s > 0.05; marginal R^2^ = 0.061, conditional R^2^ = 0.359) (Table 1).

### 3.2. Principal Component Analysis of Fixation Allocation

The PCA of fixation proportions yielded five retained components explaining 70.9% of the variance in total, with individual explained variances of 21.7%, 15.7%, 11.9%, 11.0%, and 10.7% for components 1 to 5, respectively. Sampling adequacy for the fixation data was moderate (KMO = 0.57), and Bartlett’s test of sphericity was significant, indicating that the data were suitable for exploratory component extraction.

The PCA of duration proportions likewise yielded five retained components, explaining 64.5% of the total variance, with individual explained variances of 18.2%, 13.3%, 12.5%, 10.4%, and 10.2%. For the duration data, Bartlett’s test of sphericity could not be computed due to a near-singular correlation matrix, reflecting strong interdependence among AOI variables inherent to proportional data. Sampling adequacy was lower (KMO = 0.52), and component structure should therefore be interpreted as indicative of general patterns in gaze allocation rather than as well-defined latent dimensions.

Varimax-rotated loadings indicated that the components reflected contrasts in attentional allocation rather than isolated AOIs. For fixation, FRC1 was characterized by positive loading on Ears and Eyes (0.58) and negative loadings on Rider leg (−0.56) and Horse’s shoulder (−0.51), while FRC2 contrasted Mouth (−0.65) with Rider shoulder (0.56). FRC3 was dominated by Neck (0.76) and Rider head (−0.59), FRC4 by Front legs (0.52) and Rider pelvis (−0.65), and FRC5 by Croup (0.66) and Hindlegs (0.55).

For duration, DRC1 contrasted Ears and Eyes (−0.61) and Mouth (−0.61), with smaller positive loadings on Front Legs (0.35). DRC2 was dominated by Rider pelvis (−0.59) with positive contributions from Hindlegs (0.42) and Front Legs (0.38). DRC3 contrasted Croup (0.73) with Horse’s shoulder (−0.60), DRC4 was dominated by Rider head (−0.82), and DRC5 contrasted Rider shoulder (0.61) with Rider leg (−0.62) (Table 2).

### 3.3. Correlations Among Gaze Strategies

**Figure 2:**
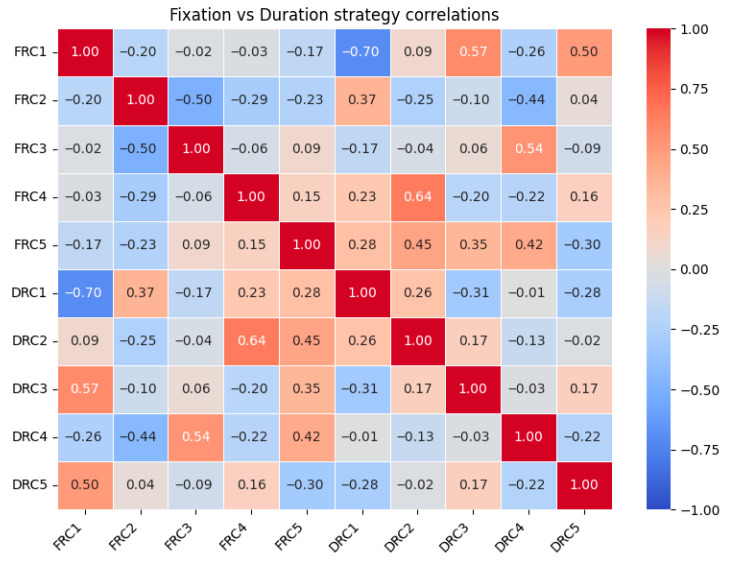
Pearson correlation matrix of fixation-based (FRC) and duration-based (DRC) gaze strategy components. Warmer colors indicate positive correlations, and cooler colors indicate negative correlations. Several strong cross-modal correlations are evident (e.g., FRC1–DRC1, FRC4–DRC2) alongside weaker associations, indicating partial overlap but also distinct patterns of visual attention allocation.

Pearson’s correlations between fixation-based and duration-based components indicated a mixture of strong, moderate, and weak cross-modal relationships. While some components showed substantial overlap (e.g., FRC1–DRC1: r = −0.70, *p* < 0.001; FRC4–DRC2: r = 0.64, *p* < 0.001), many others were only weakly related (e.g., FRC4–DRC3: r = −0.20, *p* = 0.30) (Figure 2; Appendix A, Table A1).

These patterns suggest that fixation counts and duration measures share variance in certain dimensions, while also capturing partially distinct aspects of visual attention allocation. This supports their inclusion as complementary predictors within the same inferential model.

### 3.4. Predicting Harmony Scores

All LMMs converged successfully without warnings. Visual inspection of residuals against fitted values did not indicate systematic deviations from homoscedasticity or linearity, and residual distributions were approximately normal. Given the use of orthogonal PCA-derived predictors, no problematic multicollinearity was expected, which was confirmed in diagnostic checks.

A LMM on the effect of the five fixation strategies and the five duration strategies on harmony scores showed that participants rated the *eventing* video significantly lower in harmony compared to the *dressage* reference video (*B* = −0.85, *SE* = 0.39, *z* = −2.17, *p* < 0.05). Participant experience level (Professional vs. Amateur) did not significantly predict harmony scores (*B* = −0.13, *SE* = 0.46, *z* = −0.27, *p* = 0.79).

**Figure 3:**
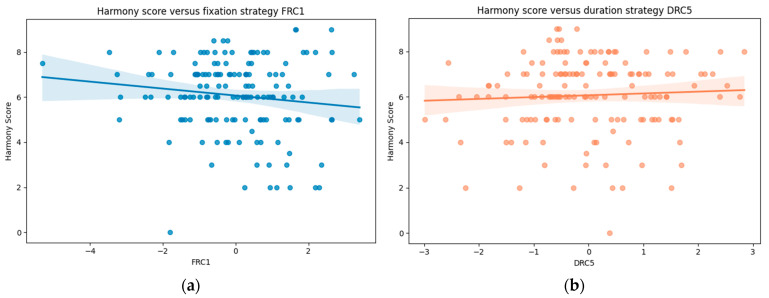
(**a**) Relationship between fixation strategy FRC1 and harmony scores. The negative slope indicates that greater fixation on the horse’s ears and eyes relative to rider leg and horse shoulder (FRC1) were associated with lower harmony scores. (**b**) Relationship between duration strategy DRC5 and harmony scores. The positive slope indicates that longer dwell time on the rider’s shoulder relative to rider leg (DRC 5) was associated with higher harmony scores. Shaded areas represent 95% confidence intervals.

Controlling for video and experience level, a significant negative association between FRC1 and harmony scores was (*B* = −0.34, *SE* = 0.13, *z* = −2.53, *p* < 0.05), indicating that a greater focus on the horse’s Ears and Eyes (and away from Rider leg) was associated with lower harmony scores (Figure 3). A significant positive association was also observed between DRC5 and harmony scores (B = 0.25, SE = 0.12, z = 2.12, *p* < 0.05), indicating that relatively longer fixation durations on Rider shoulder (and shorter focus on Rider leg) were associated with higher harmony scores (Figure 3). Model fit indices indicated a marginal R^2^ of 0.120 and a conditional R^2^ of 0.440, indicating that fixed effects explained 12.0% of variance in harmony scores, with between-participant differences accounting for an additional 32.0%.

Given the lower sampling adequacy of the duration-based PCA, this finding should be interpreted with appropriate caution.

No other fixation or duration components significantly predicted harmony scores (all *p*s > 0.10) (Table 3).

### 3.5. Variation and Interrelations of Gaze Strategies

Categorization of participants by their single dominant fixation and duration strategy (based on their highest absolute component score) showed that dominant fixation strategies varied across individuals, with FRC1 most frequently dominant (*n* = 10), followed by FRC4 (*n* = 9), FRC3 (*n* = 5), FRC5 (*n* = 4), and FRC2 (*n* = 2). Dominant duration strategies were more evenly distributed, with DRC4 most frequent (*n* = 8), followed by DRC3 (*n* = 7), DRC1 (*n* = 7), DRC2 (*n* = 6), and DRC5 (*n* = 2) (Appendix A, Table A2).

While the effect of video discipline remained consistent across models (with eventing continuing to predict lower harmony scores), LMMs indicated no significant main effects of possessing any specific dominant fixation strategy or duration strategy when compared to the reference groups (all *p*s > 0.05; marginal R^2^ = 0.067 and conditional R^2^ = 0.375 for the fixation model; marginal R^2^ = 0.072 and conditional R^2^ = 0.395 for the duration model) (Table 4 and Table 5).

### 3.6. Qualitative Conceptualization of Harmony

Thematic analysis identified thirteen lower-order themes grouped into three higher-order themes: Horse Behavior, Rider Influence, and Horse–Rider Connection.

The Horse Behavior theme comprised equine facial expressions, horse’s emotion, horse’s reaction, movement, and tension and relaxation, capturing how participants described the horse’s behavioral expression, responsiveness, and apparent tension or relaxation.

Rider Influence comprised aid intensity, rider aids, and rider tension, reflecting how participants described the role of the rider in shaping performance, particularly in terms of clarity, strength and appropriateness of aids, as well as physical tension or control. Horse–Rider Connection comprised communication, degree of difficulty, emotional bond, overall impression and unity, representing more integrative judgments of the interaction between horse and rider, including perceived harmony, coordination, and the overall quality of the partnership.

Total mentions were highest for Horse Behavior (*n* = 58), followed by Horse–Rider Connection (*n* = 42) and Rider Influence (*n* = 34). A more detailed description of the content of the lower order themes can be found in Appendix B, Table A3. At participant level, the three theme proportions summed to 1.00 across all cases (mean = 1.00, SD = 2.92 × 10^−17^), confirming correct normalization. Participants varied in the relative emphasis placed on the three higher-order themes, with some distributing their references relatively evenly across themes and others placing greater emphasis on a single domain (see Appendix A, Table A1).

Exploratory LMMs on participants’ qualitative conceptualization of harmony on quantitative scoring indicated that the qualitative themes did not significantly predict harmony scores in either model, with marginal R^2^ = 0.080 and conditional R^2^ = 0.424 for the duration-based model and marginal R^2^ = 0.093 and conditional R^2^ = 0.419 for the fixation-based model. In the duration-based model, neither Horse Behavior (B = −0.67, SE = 0.89, z = −0.75, *p* = 0.45) nor Rider Influence (B = −0.45, SE = 1.31, z = −0.34, *p* = 0.73) were significantly associated with the scores awarded (see Table 6 and Table 7). Identical non-significant results were observed in the fixation-based model for both Horse Behavior (B = −0.21, SE = 0.88, z = −0.23, *p* = 0.82) and Rider Influence (B = −0.35, SE = 1.30, z = −0.27, *p* = 0.79). 

## 4. Discussion

The current study explored how equestrians conceptualize horse–rider harmony and how these conceptualizations relate to visual attention and subsequent evaluation. Two key findings emerged. First, participants demonstrated a consistent and structured conceptual understanding of harmony. Second, visual inspection was organized into distinct higher-order gaze strategies, with two strategies showing significant associations with harmony scores. 

At the conceptual level, participants mentioned aspects relevant to the higher-order theme of Horse Behavior most frequently, indicating that how the horse responds is considered essential in the assessment of harmony. More specifically, equine facial expressions, such as ear position and tension around the eyes, mouth and nostrils were considered important indicators, closely aligning with previous research on the importance of equine facial expressions and body language in shaping human assessment of horses [36,37,38]. Horses that appeared “happy” or “relaxed,” were also thought to embody harmony, demonstrating the inclination of people to draw on anthropomorphic terminology to express perceptions of harmony [36]. Conflict signs such as an open mouth, tail swishing, or stiffness were frequently cited as evidence of miscommunication or resistance, while prompt reactions and relaxed movement were associated with harmony. Such findings align closely with previous research that links conflict behaviors to poor rider balance or inconsistent cues [20,39].

The higher-order theme of Horse–Rider Connection captured the relational dimension of harmony, encompassing unity, mutual understanding, and coordinated movement between horse and rider. Participants described harmonious interactions as moments in which the dyad appeared to “move as one” or to “think the same thought,” capturing the idea that harmony might be both a reflection of biomechanical synchronization [11,12,13,14,40] as well as a form of non-verbal dialog within the dyad [6,41].

Within the theme of Rider Influence, the contribution of the rider to a harmonious interaction was consistently described as subtle, with well-timed, minimally visible aids and a stable, independent seat forming the basis for effective interaction, in line with descriptions of “feel” as an embodied and experience-based skill [6,15].

Together, these findings indicate that participants share a coherent and multidimensional conceptual framework of harmony, consistent with both the biomechanical and psychological perspectives described in the literature [14,20,28]. However, the absence of significant associations between qualitative theme proportions and harmony scores suggests that this conceptual framework does not automatically translate into a repeatable scoring of harmony. Such an interpretation is further supported by the model fit indices, which indicate that harmony assessment is neither entirely idiosyncratic nor fully standardized. Even in a group as diverse as the study participants, the fixed predictors including gaze strategies explain a meaningful proportion of variance, pointing to a shared perceptual understanding of harmony. At the same time, the greater proportion of variance explained by between-participant differences underscores the role of individual perceptual tendencies in shaping harmony assessment.

At a conceptual level, therefore, harmony appears to be a shared construct. In its practical application, however, it is personally enacted. This may provide an explanation for the persistent variability observed in equestrian judging, where even formally trained judges applying the same criteria can diverge considerably in their scores [42]. Such variability is consistent with what Kahneman et al. [43] describe as ‘noise’, namely the random variation in judgment that occurs even among individuals who share the same conceptual framework and are ostensibly applying the same criteria. Heuristics and cognitive biases further compound this variability, as judges operating under time pressure and cognitive load inevitably rely on perceptual shortcuts that reflect individual experience and preference as much as formal criteria [27,42]. Nevertheless, findings did demonstrate coherent structuring of visual attention into identifiable and interpretable patterns of gaze allocation. These fixation-based and duration-based components reflect the structured distribution of attention across anatomically and functionally related regions. While previous equestrian eye tracking research has relied on a priori clustering of areas of interest to reflect broader regions such as the front or back of the horse, feet and rider [28,42], the current study demonstrates that clustering of smaller anatomical areas of interest allows for a more specific identification of gaze strategies. As has been shown by previous research investigating the relationship between visual search behavior and expertise across different sports, such as squash [30], gymnastics [31], rugby [33], handball [44] and artistic swimming [45], fine-grained perceptual cues underpin performance and evaluation.

More specifically, two gaze strategies emerged as significant predictors of harmony scores. For one, the number of fixations on the horse’s ears and eyes relative to the rider’s leg and the horse’s shoulder was associated with lower harmony scores. The horse’s facial region may function as a diagnostically relevant area to which observers return when evaluating behavioral expression. Frequent (re)visits to this region may reflect the ongoing assessment of cues relating to signs indicative of the horse’s mental state, such as tension, alertness or relaxation. Increased scrutiny might be indicative of mounting doubt about perceived levels of harmony, ultimately resulting in lower harmony scores. Conversely, fewer fixations on equine facial features, and correspondingly more frequent focus on the more mechanistic aspects of riding evidenced by the movement of the shoulder and the positioning of the rider’s leg was associated with greater harmony scores. In the absence of disconcerting facial cues, attention likely shifts towards movement quality and rider influence, resulting in a more favorable harmony assessment.

What is more, this particular gaze strategy was also the most prominent across participants, with the horse’s ears and eyes as the most frequent first fixation location. Human perception research has shown that observers tend to fixate on faces, and particularly the eye region, when extracting socially relevant information [46,47,48,49]. Similar mechanisms may operate in horse–human contexts, where the horse’s facial features function as a visual anchor for interpreting affective and behavioral state [38,50]. As such, current findings underscore the importance of equine facial expressions when gauging the quality of horse-human relationships, and, by extension, equine welfare [20,37]. However, this also highlights the importance of accurate recognition and interpretation of facial features in equestrian practice, seeing that both over- and under-estimation of equine facial expressions can have serious consequences [38,51,52].

At the same time, a duration-based gaze strategy characterized by a longer dwell time on the rider’s shoulder relative to their leg was associated with higher harmony scores. Sustained attention to the rider’s upper body might be indicative of assessing rider balance, coordination, and communication within the dyad. Balance, in this context, refers to the rider’s biomechanical stability and independence of seat; a quality that enables the rider to move with the horse without interference, creating the conditions for subtle and responsive interaction [15]. This may be particularly relevant in situations where the interaction appears stable and requires less repeated observation of behavioral cues. In this sense, variation in gaze allocation can be interpreted in relation to evaluative demand, with certain regions attracting repeated attention when additional information is required. However, given the greater interdependence of variables in the duration-based PCA, these patterns should be interpreted with appropriate caution.

Beyond the significant predictors, the broader pattern of gaze strategies across participants revealed further insights. Previous eye tracking research in sport has often reported differences between expertise levels, with more experienced judges demonstrating more efficient and selective visual search strategies [30,31,32]. In the current study, participant experience level did not significantly predict harmony scores across disciplines. However, all participants had considerable practical experience with horses. Seeing that the harmonious interaction between horse and rider is inherent to equestrian sports [6,13,16,17], it may be argued that participants were all able to access and interpret task-relevant cues, enabling the use of similar perceptual strategies. Haider and French (1999) [53] propose in their information reduction hypothesis that individuals with domain-specific experience process information more efficiently by focusing on relevant cues while filtering out less informative input. Within the context of horse–rider evaluation, the horse’s facial expressions and the rider’s primary coordination system likely function precisely as such clues, recognizable by both professionals and non-professionals with relevant experience. A detailed comparison between participant categories such as judges and trainers was beyond the scope of the present study, given the small subgroup sizes. However, research comparing observer groups in esthetic sports suggests that expertise level and domain-specific experience drive visual search differences more robustly than formal role, with gaze patterns converging when observers share equivalent experiential knowledge of the task [31], supporting the current study’s categorization of participants by experience level rather than professional role.

The distinction between fixation-based and duration-based strategies further highlights the multidimensional nature of visual attention. While some components showed substantial overlap across modalities, others remained distinct, indicating that fixation frequency and dwell time capture complementary aspects of perceptual processing [25]. This distinction is relevant for understanding perceptual expertise, as it reflects differences not only in where observers look, but also in how visual information is processed over time. 

Interestingly, eventing was consistently given lower harmony scores compared to the other disciplines. The video in question showed the cross-country phase, which in general is characterized through higher speeds and thus greater physical exertion than other disciplines [54,55]. Previous research has indicated that physiological stress, such as exercise, may produce facial changes that overlap with, and are hard to distinguish from, indicators of discomfort [39,56,57]. This might mean that participants could have interpreted facial signals as indicative of mental stress, while in reality, this could merely have been a reflection of physical exertion.

Taken together, the findings indicate that the relationship between conceptualization, perception, and evaluation is mediated by an additional interpretative layer, which must be taken into account. Perceptual input is integrated with experience, implicit expertise inherent to the nature of equestrianism, and contextual expectations [27,29]. So, even though participants share a common understanding of harmony at the conceptual level and a number of coherent gaze strategies, only two of these strategies predicted harmony scores. The other gaze strategies diverged considerably among participants, with no direct influence on harmony assessment. While these findings support the idea of harmony as a construct that is both shared and individually enacted, they also show yet again that this does not automatically lead to consistent evaluation in practice. For equestrian sport, where harmony is a formal assessment criterion [8,9,10], this has direct relevance for transparency and consistency in judging.

Further development of explicit and shared assessment frameworks for harmony may support more consistent evaluation and provide clearer guidance for riders and judges. In addition, increasing insight into how harmony is visually assessed may contribute to strengthening the sector’s social license to operate by demonstrating a credible and welfare-oriented approach to performance evaluation [22,28,42].

### Limitations

Several limitations should be acknowledged. First, the relatively high exclusion rate of 47% reduced the final sample size to 30 participants. While exclusions were based solely on objective technical quality criteria and are therefore unlikely to have introduced systematic bias into the retained sample, the reduced sample size limits statistical power and the generalizability of findings. The exploratory nature of the study and the practical constraints of collecting high-quality eye tracking data in applied settings further contributed to this limitation. Future studies would benefit from more controlled lighting conditions and equipment less sensitive to ambient light variation. While the number of observations at the participant-by-video level was sufficient for the mixed-effects analyses, the limited number of participants should be kept in mind when interpreting the findings.

Second, the video clips could not be randomized due to technical constraints, and each discipline was represented by a single clip. The fixed presentation order may have introduced order effects. However, classical anchoring effects in sequential judgment require a common evaluative dimension across stimuli, such that earlier performances serve as implicit reference points for later ones [42]. In the current study, each clip depicted a different discipline performing a fundamentally different movement, making direct cross-stimulus comparison considerably less meaningful. The diversity of disciplines depicted, combined with the use of a single clip per discipline, further reduces the likelihood of anchoring, as there is no common performance dimension on which participants could meaningfully base comparative judgements. The only shared evaluative dimension across all clips was harmony itself, meaning that any consistent perceptual response to harmony-relevant cues across disciplines would constitute a finding rather than a bias. The absence of systematic trends in harmony scores across the viewing sequence further supports this interpretation. The use of a single clip per discipline does, however, limit the generalizability of discipline-specific findings, and future studies would benefit from including multiple clips per discipline and randomizing presentation order where technically feasible. In addition, fixation behavior may have been influenced by factors unrelated to harmony assessment, such as video resolution, background context, or discipline-specific movement characteristics.

Third, the use of proportional gaze data introduces inherent interdependence between variables, which constrained certain statistical diagnostics and requires cautious interpretation of component structure.

Finally, two observations were excluded from the first fixation analysis due to missing or invalid first fixation data, resulting in a slightly reduced sample (*n* = 148) for this model. While this difference is minimal and unlikely to affect the overall pattern of results, it should be noted for completeness.

## 5. Conclusions

The present study sought to clarify the concept of horse–rider harmony by examining how equestrians define, perceive, and evaluate it. The findings indicate that participants share a coherent and multidimensional understanding of harmony, composed of equine behavior, rider influence, and their interaction. Equine behavioral expression appeared to take precedence in how harmony is assessed.

While the shared conceptual framework did not predict gaze strategies or harmony scores directly, eye tracking results indicated that participants employ coherent gaze allocation patterns aimed at specific anatomical areas. The horse’s ears and eyes emerged as particularly prominent, suggesting that the horse’s face may act as an initial anchor for interpretation. This aligns with broader findings in human perception, where faces serve as primary entry points for social information. At the same time, variation in attention to rider-related regions, particularly the relative focus on the rider’s shoulder compared to the rider’s leg, indicates that rider cues also contribute to how harmony is perceived, likely when aspects relating to the impact of rider balance and coordination are assessed.

Taken together, the pattern of findings suggests that harmony operates as a shared construct at the conceptual level, while remaining personally enacted in its practical application. This distinction may help explain the persistent variability observed in equestrian judging, and points to the value of developing more explicit and shared assessment frameworks.

Although these findings should be interpreted with some caution, they point to the potential importance of both facial- and rider-related cues in harmony assessment. Further research is needed to examine how such cues shape evaluation and how they may be incorporated into more explicit and consistent assessment frameworks. Improving education around the recognition of behavioral and biomechanical indicators may support more transparent and welfare-oriented evaluation practices in equestrian sport.

## Figures and Tables

**Table 1:** Mixed linear model predicting harmony scores based on first fixation location.

	Coef.	Std.Err.	z	*p* > |z|	[0.03	0.98]
Intercept	6.32	0.82	7.72	0.00	4.71	7.92
FirstAOI[T.Ears and Eyes]	0.04	0.83	0.05	0.96	−1.58	1.66
FirstAOI[T.Front legs]	−0.61	1.02	−0.60	0.55	−2.61	1.40
FirstAOI[T.Mouth]	0.24	0.94	0.26	0.80	−1.61	2.09
FirstAOI[T.Neck]	0.07	0.92	0.08	0.94	−1.74	1.88
FirstAOI[T.Rider pelvis]	0.09	0.86	0.10	0.92	−1.59	1.76
FirstAOI[T.Rider head]	0.46	0.99	0.46	0.64	−1.48	2.39
FirstAOI[T.Rider leg]	0.28	0.97	0.29	0.78	−1.62	2.17
FirstAOI[T.Rider shoulder]	0.17	0.84	0.20	0.84	−1.47	1.81
FirstAOI[T.Shoulder (horse)]	0.29	0.90	0.32	0.75	−1.48	2.06
Video_label[T.Eventing]	−0.76	0.39	−1.96	0.05	−1.53	0.00
Video_label[T.Icelandic Riding]	−0.64	0.39	−1.66	0.10	−1.40	0.12
Video_label[T.Showjumping]	0.07	0.42	0.17	0.86	−0.75	0.90
Video_label[T.Working Equitation]	0.17	0.42	0.41	0.68	−0.65	0.99
Level_label[T.Professional]	−0.20	0.45	−0.45	0.65	−1.08	0.68
Group Var.	0.91	0.29				

**Table 2:** Varimax loadings for fixation (FRC) and duration (DRC) retained components.

	FRC1	FRC2	FRC3	FRC4	FRC5	DRC1	DRC2	DRC3	DRC4	DRC5
Croup	0.13	−0.10	0.04	−0.14	0.66	0.05	0.22	0.73	0.07	0
Ears and Eyes	0.58	−0.22	−0.04	0.04	0.00	−0.61	0.02	0.13	−0.13	0.14
Front legs	−0.12	0.17	0.01	0.52	0.20	0.35	0.38	−0.12	0.06	0.34
Hindlegs	−0.06	0.15	−0.10	0.24	0.55	0.22	0.42	−0.07	0.15	−0.27
Mouth	−0.01	−0.65	−0.13	−0.07	−0.20	−0.61	0.12	−0.25	0.37	−0.1
Neck	0.09	0.17	0.76	0.18	−0.22	0.06	−0.37	−0.07	−0.12	0.04
Rider pelvis	−0.19	0.23	0.03	−0.65	0.12	0.12	−0.59	0.02	0.26	−0.14
Rider head	0.10	0.19	−0.59	0.25	−0.19	−0.01	−0.06	−0.08	−0.82	−0.05
Rider leg	−0.56	−0.14	−0.06	−0.12	0.00	0.19	−0.14	−0.02	0.11	−0.62
Rider shoulder	0.04	0.56	−0.19	−0.14	−0.27	0.15	−0.28	−0.04	0.24	0.61
Shoulder (horse)	−0.51	−0.14	0.04	0.31	−0.09	0.08	0.15	−0.6	0	0.02

**Table 3:** Mixed linear model predicting harmony scores from fixation and duration strategies.

	Coef.	Std.Err.	z	*p* > |z|	[0.03	0.98]
Intercept	6.36	0.46	13.97	0.00	5.47	7.25
Video_label[T.Eventing]	−0.85	0.39	−2.17	0.03	−1.62	−0.08
Video_label[T.Icelandic Riding]	−0.38	0.39	−0.98	0.33	−1.15	0.38
Video_label[T.Showjumping]	−0.02	0.42	−0.04	0.97	−0.84	0.80
Video_label[T.Working Equitation]	0.21	0.42	0.50	0.62	−0.62	1.04
Level_label[T.Professional]	−0.13	0.46	−0.27	0.79	−1.03	0.78
FRC1	−0.34	0.13	−2.53	0.01	−0.60	−0.08
FRC2	0.06	0.12	0.45	0.65	−0.19	0.30
FRC3	−0.11	0.13	−0.81	0.42	−0.36	0.15
FRC4	−0.14	0.13	−1.12	0.26	−0.39	0.11
FRC5	−0.17	0.14	−1.18	0.24	−0.44	0.11
DRC1	−0.16	0.14	−1.13	0.26	−0.44	0.12
DRC2	0.14	0.12	1.20	0.23	−0.09	0.37
DRC3	0.06	0.12	0.51	0.61	−0.17	0.29
DRC4	−0.08	0.13	−0.59	0.55	−0.33	0.18
DRC5	0.25	0.12	2.12	0.03	0.02	0.48
Group Var.	0.10	0.31				

**Table 4:** Mixed linear model predicting harmony scores from dominant fixation strategy.

	Coef.	Std.Err.	z	*p* > |z|	[0.03	0.98]
Intercept	6.36	0.49	13.10	0.00	5.41	7.32
C(DominantFixStrategy)[T.FRC2]	0.02	0.39	0.06	0.95	−0.73	0.78
C(DominantFixStrategy)[T.FRC3]	0.50	0.37	1.33	0.18	−0.24	1.23
C(DominantFixStrategy)[T.FRC4]	0.08	0.42	0.19	0.85	−0.74	0.90
C(DominantFixStrategy)[T.FRC5]	0.003	0.36	0.01	0.99	−0.69	0.70
Video_label[T.Eventing]	−0.73	0.36	−2.04	0.04	−1.43	−0.03
Video_label[T.Icelandic Riding]	−0.60	0.36	−1.70	0.09	−1.30	0.09
Video_label[T.Showjumping]	0.006	0.36	0.02	0.99	−0.70	0.71
Video_label[T.Working Equitation]	0.20	0.35	0.56	0.57	−0.49	0.89
Level_label[T.Professional]	−0.27	0.44	−0.62	0.54	−1.14	0.59
Group Var.	0.91	0.28				

**Table 5:** Mixed linear model predicting harmony scores from dominant duration strategy.

	Coef.	Std.Err.	z	*p* > |z|	[0.03	0.98]
Intercept	6.32	0.51	12.45	0.00	5.32	7.31
C(DominantDurStrategy)[T.DRC2]	−0.08	0.39	−0.19	0.85	−0.84	0.69
C(DominantDurStrategy)[T.DRC3]	0.22	0.38	0.57	0.57	−0.53	0.97
C(DominantDurStrategy)[T.DRC4]	0.61	0.42	1.47	0.14	−0.20	1.42
C(DominantDurStrategy)[T.DRC5]	0.03	0.39	0.07	0.95	−0.75	0.80
Video_label[T.Eventing]	−0.80	0.35	−2.28	0.02	−1.48	−0.11
Video_label[T.Icelandic Riding]	−0.60	0.35	−1.70	0.09	−1.29	0.09
Video_label[T.Showjumping]	0.01	0.35	0.03	0.98	−0.68	0.70
Video_label[T.Working Equitation]	0.21	0.35	0.58	0.56	−0.49	0.90
Level_label[T.Professional]	−0.23	0.45	−0.52	0.60	−1.11	0.65
Group Var.	0.97	0.30				

**Table 6:** Mixed linear model predicting harmony scores from duration strategies and qualitative theme proportions.

	Coef.	Std.Err.	z	*p* > |z|	[0.03	0.98]
Intercept	7.00	0.75	9.39	0.00	5.54	8.46
Video_label[T.Eventing]	−0.88	0.38	−2.33	0.02	−1.63	−0.14
Video_label[T.Icelandic Riding]	−0.70	0.36	−1.97	0.05	−1.40	−0.002
Video_label[T.Showjumping]	−0.10	0.37	−0.26	0.80	−0.82	0.63
Video_label[T.Working Equitation]	0.13	0.39	0.33	0.74	−0.63	0.88
Level_label[T.Professional]	−0.36	0.48	−0.75	0.45	−1.30	0.58
DRC1	−0.03	0.12	−0.25	0.80	−0.27	0.21
DRC2	0.07	0.11	0.64	0.52	−0.14	0.28
DRC3	−0.01	0.12	−0.05	0.96	−0.23	0.22
DRC4	−0.01	0.12	−0.10	0.92	−0.25	0.22
DRC5	0.21	0.11	1.91	0.06	−0.01	0.42
HorseProp	−0.67	0.89	−0.75	0.45	−2.41	1.08
RiderProp	−0.45	1.31	−0.34	0.73	−3.02	2.12
Group Var.	1.08	0.33				

**Table 7:** Mixed linear model predicting harmony scores from fixation strategies and qualitative theme proportions.

	Coef.	Std.Err.	z	*p* > |z|	[0.03	0.98]
Intercept	6.56	0.74	8.89	0.00	5.11	8.00
Video_label[T.Eventing]	−0.82	0.38	−2.18	0.03	−1.56	−0.08
Video_label[T.Icelandic Riding]	−0.42	0.37	−1.13	0.26	−1.14	0.31
Video_label[T.Showjumping]	0.11	0.38	0.30	0.77	−0.63	0.85
Video_label[T.Working Equitation]	0.31	0.41	0.74	0.46	−0.51	1.12
Level_label[T.Professional]	−0.23	0.47	−0.49	0.62	−1.15	0.69
FRC1	−0.19	0.12	−1.63	0.10	−0.41	0.04
FRC2	0.04	0.12	0.36	0.72	−0.19	0.28
FRC3	−0.09	0.13	−0.67	0.50	−0.34	0.16
FRC4	−0.09	0.12	−0.77	0.44	−0.32	0.14
FRC5	−0.19	0.13	−1.54	0.12	−0.44	0.05
HorseProp	−0.21	0.88	−0.23	0.82	−1.93	1.52
RiderProp	−0.35	1.30	−0.27	0.79	−2.89	2.19
Group Var.	1.02	0.32				

## Data Availability

The data presented in this study are openly available in DANS Data Station Life Sciences at https://doi.org/10.17026/LS/YSSHLS.

## Appendix A

**Table A1:** Pearson correlation coefficients (r) and associated *p*-values for all pairwise combinations of fixation-based (FRC) and duration-based (DRC) gaze strategy components.

	FRC	DRC	r	*p*
0	FRC1	DRC1	−0.70	0.000
1	FRC1	DRC2	0.09	0.644
2	FRC1	DRC3	0.57	0.001
3	FRC1	DRC4	−0.26	0.172
4	FRC1	DRC5	0.50	0.005
5	FRC2	DRC1	0.37	0.047
6	FRC2	DRC2	−0.25	0.187
7	FRC2	DRC3	−0.10	0.609
8	FRC2	DRC4	−0.44	0.014
9	FRC2	DRC5	0.04	0.831
10	FRC3	DRC1	−0.17	0.369
11	FRC3	DRC2	−0.04	0.837
12	FRC3	DRC3	0.06	0.763
13	FRC3	DRC4	0.54	0.002
14	FRC3	DRC5	−0.09	0.644
15	FRC4	DRC1	0.23	0.220
16	FRC4	DRC2	0.64	0.000
17	FRC4	DRC3	−0.20	0.295
18	FRC4	DRC4	−0.22	0.248
19	FRC4	DRC5	0.16	0.401
20	FRC5	DRC1	0.28	0.127
21	FRC5	DRC2	0.45	0.013
22	FRC5	DRC3	0.35	0.060
23	FRC5	DRC4	0.42	0.021
24	FRC5	DRC5	−0.30	0.107

**Table A2:** Individual dominant fixation (FRC) and duration (DRC) strategies across participants.

Participant	Category	Level	DRC1	DRC2	DRC3	DRC4	DRC5	DominantDur	FRC1	FRC2	FRC3	FRC4	FRC5	DominantFix	HorseProp	RiderProp	ConnectionProp
2	Horse Owner	Amateur	0.08	0.69	−0.54	1.16	−0.86	DRC4	−1.10	−1.16	0.51	0.47	1.35	FRC5	0.56	0.11	0.33
4	Rider	Amateur	0.07	−0.38	−1.06	−1.48	0.16	DRC4	−0.81	0.54	−1.69	0.57	−1.38	FRC3	0.20	0.40	0.40
5	Trainer	Professional	−0.07	−0.81	−0.77	0.93	−0.08	DRC4	−0.59	−0.20	0.93	−0.04	−0.88	FRC3	0.50	0.33	0.17
9	Education	Professional	−0.03	0.32	−1.13	−0.56	0.58	DRC3	0.16	0.38	−0.54	0.31	−0.97	FRC5	0.20	0.40	0.40
13	Trainer	Professional	0.54	1.24	−0.55	−0.56	−0.26	DRC2	0.53	0.23	−0.12	1.04	0.03	FRC4	0.40	0.20	0.40
14	Trainer	Professional	0.22	−0.81	−0.45	0.35	−0.80	DRC2	−0.75	0.22	1.27	−0.34	−0.44	FRC3	0.50	0.00	0.50
15	Trainer	Professional	−0.24	0.43	0.46	−0.81	−0.42	DRC4	0.80	−0.41	−0.15	−0.19	−0.13	FRC1	0.50	0.50	0.00
16	Rider	Amateur	−1.22	0.23	0.97	0.38	−0.24	DRC1	0.46	−0.29	0.29	−0.64	0.33	FRC4	0.60	0.20	0.20
17	Trainer	Professional	−0.19	0.10	1.40	−0.40	0.89	DRC3	0.99	−0.22	0.03	−0.12	0.03	FRC1	0.80	0.20	0.00
19	Trainer	Professional	0.28	0.01	−0.54	0.28	0.48	DRC3	−0.60	−0.44	0.08	0.78	0.61	FRC4	1.00	0.00	0.00
24	Journalist	Professional	−0.91	−0.15	1.80	0.24	0.03	DRC3	1.81	0.30	0.14	−0.59	0.35	FRC1	0.67	0.17	0.17
26	Horse Owner	Amateur	0.86	−0.69	−0.28	0.26	−0.19	DRC1	−1.05	1.15	−0.43	−1.72	−0.26	FRC4	0.33	0.33	0.33
28	Trainer	Professional	0.00	0.37	1.04	0.80	0.38	DRC3	0.98	−0.60	0.67	−0.15	0.87	FRC1	0.20	0.20	0.60
33	Horse Owner	Amateur	−1.75	0.21	−0.02	−0.30	0.34	DRC1	1.30	−0.81	1.22	0.49	−0.35	FRC1	0.67	0.00	0.33
34	Trainer	Professional	0.30	0.31	0.02	−0.61	0.46	DRC4	0.89	0.00	−0.79	0.91	−0.56	FRC4	0.00	0.50	0.50
41	Education	Professional	−0.34	−1.08	0.51	0.62	−0.12	DRC2	0.11	0.67	−0.88	−1.04	0.36	FRC4	0.50	0.50	0.00
42	Rider	Amateur	0.35	0.74	0.42	−0.64	0.01	DRC2	−0.41	0.46	0.51	0.43	0.21	FRC3	0.33	0.67	0.00
44	Trainer	Professional	−0.33	0.48	0.53	0.21	0.16	DRC3	1.40	−0.52	−0.50	0.21	1.24	FRC1	0.56	0.22	0.22
45	Horse Owner	Amateur	0.46	0.60	0.58	−0.86	0.56	DRC4	0.58	0.53	0.36	0.15	−0.41	FRC1	0.50	0.17	0.33
46	Rider	Amateur	−0.11	0.21	0.73	−1.17	−0.38	DRC4	0.66	0.32	−1.30	−0.21	−0.50	FRC3	0.33	0.33	0.33
49	Horse Owner	Amateur	−0.36	−1.33	−0.46	−0.40	−0.45	DRC2	0.38	−0.04	0.41	−1.40	−0.54	FRC4	0.60	0.20	0.20
50	Horse Owner	Amateur	−0.65	−0.85	0.09	0.59	0.91	DRC5	−0.14	−0.65	0.60	−0.24	−0.57	FRC2	0.50	0.25	0.25
51	Judge	Professional	0.41	−0.50	−0.90	0.62	0.36	DRC3	−0.92	0.67	0.33	−1.09	−0.45	FRC4	0.00	0.33	0.67
52	Judge	Professional	1.34	−0.25	−0.17	−1.01	−0.11	DRC1	−1.73	1.38	−0.92	0.59	1.13	FRC1	0.00	0.00	1.00
54	Judge	Professional	−1.50	−0.99	−0.69	−0.20	0.13	DRC1	0.89	0.09	−0.55	−0.41	−1.07	FRC5	0.00	0.00	1.00
55	Judge	Professional	0.13	−0.19	0.43	0.44	−0.44	DRC4	−0.21	−0.72	0.41	−0.06	0.25	FRC2	0.20	0.40	0.40
56	Judge	Professional	0.20	0.90	0.02	0.39	0.20	DRC2	0.24	0.23	−0.70	0.37	0.81	FRC5	0.17	0.33	0.50
57	Judge	Professional	1.34	0.77	−0.67	0.79	−1.20	DRC1	−2.90	−0.46	0.06	0.11	0.93	FRC1	0.00	0.00	1.00
58	Judge	Professional	0.35	0.37	−0.44	0.25	0.51	DRC5	0.41	−0.62	0.27	1.20	−0.25	FRC4	0.33	0.33	0.33
59	Judge	Professional	0.77	0.05	−0.31	0.70	−0.58	DRC1	−1.36	−0.03	0.48	0.61	0.28	FRC1	0.80	0.20	0.00

## Appendix B

**Table A3:** Descriptions of the higher-order and lower-order themes defining harmony between horse and rider as identified through inductive qualitative analysis in NVivo 15.

Lower-Order Theme	Description	Total Mentions
Higher-order theme: Horse Behavior		58
Equine Facial Expressions	This subcategory refers to visible expressions in the horse’s face, such as ear position and tension in eyes, mouth, and nostrils.	10
Horse’s Emotion	This subcategory captures explicit references to the horse’s emotional state, including terms like happy, stressed, content, or satisfied. Participants infer emotional experience from both physical cues and overall demeanor.	13
Horse’s Reaction	This subcategory includes comments on how the horse responds to the rider’s aids. It covers promptness and conflict behaviors (e.g., tail swishing, mouth activity, resisting).	18
Movement	This subcategory addresses the horse’s way of moving, including the use of its body (e.g., head–neck position, movement through the back, rhythm, suppleness).	7
Tension and Relaxation	This subcategory reflects how participants interpret muscular and emotional tension in the horse. Tension is acknowledged as part of performance, but its quality and management are important. Positive tension is often contrasted with excessive or negative tension unless followed by visible relaxation.	10
Higher-order theme: Rider Influence		34
Aid Intensity	This subcategory refers to the strength and visibility of the rider’s aids. Participants often value subtle, nearly invisible communication, where the horse appears to respond as if on its own. Overuse or harsh application of reins or legs is typically viewed as disruptive to harmony.	18
Rider Aids	This subcategory includes references to the rider’s use of rein, leg, and seat aids. It focuses on how these aids are applied and coordinated to influence the horse’s movement and posture. Comments in this area often assess the rider’s technical skill and clarity in giving aids.	15
Rider Tension	This subcategory captures observations of muscular or mental tension in the rider. A relaxed, confident rider is typically associated with better harmony and influence over the horse.	1
Higher-order theme: Horse–Rider Connection		42
Communication	This subcategory reflects how well the horse and rider understand and respond to one another. It includes mutual respect, mental connection, clear contact, and mutual understanding—often described as “having the same thoughts”.	18
Degree of Difficulty	This subcategory addresses the challenge level of the task performed relative to the horse’s training. It considers whether movements are appropriate for the horse’s capabilities.	3
Emotional Bond	This subcategory captures the perceived relationship between horse and rider. Key aspects include trust, emotional connection, and whether the horse and rider seem like a good match.	5
Overall Impression	This subcategory represents the viewer’s holistic perception of the horse–rider combination. It includes emotional responses, esthetic appreciation, and whether the performance appears pleasant to watch.	5
Unity	This subcategory relates to the harmony and coordination between horse and rider. It includes synchronization of movements, mutual rhythm, and teamwork, where horse and rider appear to move as one.	11
